# P-465. Melanocortin-4 Receptor Gene Variants and Weight Change Following Switch to Integrase Inhibitor-containing Antiretroviral Therapy

**DOI:** 10.1093/ofid/ofae631.664

**Published:** 2025-01-29

**Authors:** Todd Hulgan, Kristine M Erlandson, Yuki Bradford, Katherine Tassiopoulos, Kunling Wu, Sara H Bares, Todd T Brown, Jordan Lake, Michael A Leonard, Grace A McComsey, Marylyn Ritchie, Paul E Sax, John R Koethe, David Haas

**Affiliations:** Vanderbilt University Medical Center, Nashville, Tennessee; University of Colorado Anschutz Medical Campus, Aurora, CO; University of Pennsylvania, College Park, Pennsylvania; Harvard University T H Chan School of Public Health, Boston, Massachusetts; Harvard University T H Chan School of Public Health, Boston, Massachusetts; University of Nebraska Medical Center, Omaha, Nebraska; Johns Hopkins, Baltimore, Maryland; University of Texas Health Science Center at Houston, Houston, TX; Vanderbilt University Medical Center, Nashville, Tennessee; Case Western Reserve University, Cleveland, OH; University of Pennsylvania, College Park, Pennsylvania; Brigham and Women’s Hospital; Harvard Medical School, Boston, MA; Vanderbilt University Medical Center, Nashville, Tennessee; Vanderbilt University Medical Center, Nashville, Tennessee

## Abstract

**Background:**

Weight gain among persons with HIV (PWH) on integrase strand transfer inhibitors (INSTIs) is highly variable. Given the importance of the melanocortin-4 receptor (MC4R) in obesity, we examined associations between *MC4R* gene variants and weight gain following switch to INSTI-containing antiretroviral therapy (ART) among PWH in ACTG observational cohort studies A5001 and A5322.

**Methods:**

Eligible participants switched to their first INSTI-containing ART from 2005-2018 and had available genetic and weight data. Imputed genotypes were from genome-wide data. Linear models assessed associations between *MC4R* variants and weight change, including 20 variants previously associated with weight/obesity in populations without HIV. Analyses adjusted for age, sex at birth, genetic principal components, time after switch, and body mass index, CD4 count and virologic control at switch; separate models were stratified by race/ethnicity, sex and baseline BMI category.

**Results:**

Among 530 PWH, median age was 50 (IQR 42, 57) years, 29% were non-Hispanic Black, 22% were cis-gender female, 62% were overweight or obese at INSTI switch, and 73% switched from a protease inhibitor. INSTIs were raltegravir (62%), dolutegravir (21%), elvitegravir (17%), and bictegravir (1%). The switch regimen included tenofovir alafenamide (TAF) in 9%. Overall median weight gain after switch was 1.25 kg (IQR -1.2, 4.0). Of 20 *MC4R* variants previously associated with weight/obesity, three were nominally associated (p< 0.05) with weight change after switch in all participants or in subgroups. One (rs11873305) was associated with weight gain in all participants (p=0.01), and male (p=0.004) and White (p=0.02) subgroups. Of 507 additional *MC4R* variants, the lowest p-value among all participants was for rs56758444 (p=0.001). After Bonferroni correction, no variant was significant in *MC4R* or in exploratory genome-wide analysis.

**Conclusion:**

Among PWH, *MC4R* variants were nominally associated with weight change after switch to INSTI-based ART. These findings are consistent with the known importance of melanocortins in appetite and weight, and suggest a neuroendocrine contribution to INSTI-related weight gain. Additional studies with newer INSTIs and TAF are needed.

**Disclosures:**

**Kristine M. Erlandson, MD MS**, Gilead: Advisor/Consultant|Gilead: Grant/Research Support|ViiV: Advisor/Consultant **Sara H. Bares, MD**, Gilead Sciences: Expert Testimony|Janssen: Grant/Research Support|ViiV Healthcare: Grant/Research Support **Todd T. Brown, MD, PhD**, EMD Serono: Advisor/Consultant|Janssen: Advisor/Consultant|Merck: Advisor/Consultant|ViiV Healthcare: Advisor/Consultant|ViiV Healthcare: Honoraria **Jordan Lake, MD**, Merck: Advisor/Consultant|Theratechnologies: Advisor/Consultant **Grace A. McComsey, MD**, Gilead, Merck, GSK: Advisor/Consultant **John R. Koethe, MD**, Gilead Sciences: Advisor/Consultant|Gilead Sciences: Grant/Research Support|Merck & Co.: Advisor/Consultant|Merck & Co.: Grant/Research Support

